# First Detection of the *mcr-1* Gene in a Wild Red Fox (*Vulpes vulpes*), from a High-Density Livestock Area

**DOI:** 10.3390/antibiotics15090858

**Published:** 2026-09-02

**Authors:** Miriam Tenuzzo, Federico Scali, Claudia Romeo, Flavia Guarneri, Lucia Cioli, Silvia Filippi, Chiara Boifava, Chiara Galizia, Laura Birbes, Giovanni Parisio, Valentina Carta, Marta Masserdotti, Antonio Marco Maisano, Giovanni Loris Alborali, Benedetta Cappelletti, Lisa Guardone, Nicoletta Formenti

**Affiliations:** 1 Istituto Zooprofilattico Sperimentale della Lombardia e dell’Emilia-Romagna ‘Bruno Ubertini’ (IZSLER), 25124 Brescia, Italy; miriam.tenuzzo@izsler.it (M.T.); flavia.guarneri@izsler.it (F.G.); lucia.cioli@izsler.it (L.C.); silvia.filippi@izsler.it (S.F.); chiara.boifava@izsler.it (C.B.); chiara.galizia@izsler.it (C.G.); laura.birbes@izsler.it (L.B.); giovanni.parisio@izsler.it (G.P.); valentina.carta@izsler.it (V.C.); marta.masserdotti@izsler.it (M.M.); antoniomarco.maisano@izsler.it (A.M.M.); giovanni.alborali@izsler.it (G.L.A.); nicoletta.formenti@izsler.it (N.F.); 2 Italian Ministry of Health, 00144 Rome, Italy; b.cappelletti@sanita.it; 3 Dipartimento di Scienze Veterinarie, University of Pisa, 56124 Pisa, Italy; lisa.guardone@unipi.it

**Keywords:** Enterobacterales, multidrug resistance, ARGs, *mcr-1*, polymyxins, ESBL

## Abstract

**Background/Objectives:** Antimicrobial resistance (AMR) can be influenced by the interconnection of human, animal and environmental compartments, making wildlife-based surveillance an important component of One Health surveillance. The red fox (*Vulpes vulpes*), due to its synanthropic behaviour and wide distribution, can be a valuable indicator of environmental AMR. Mobile colistin resistance (*mcr*) genes, which encode resistance to a highest-priority critically important antimicrobial (HPCIA), have never been identified in free-ranging foxes. **Case presentation**: A red fox faecal strain isolated using selective media was PCR-screened for major AMR genes, underwent antimicrobial susceptibility testing and whole-genome sequencing (WGS). This led to the identification of the first *mcr-1*-positive *Escherichia coli* from a wild red fox. The fox inhabited an area with a small human population but one of the highest cattle densities in Italy and high antimicrobial use (AMU), as established by comparing local livestock density and AMU with surrounding areas. Despite being borderline phenotypically susceptible to colistin, the isolate displayed resistance to other HPCIAs and antimicrobial classes commonly used in livestock. WGS revealed that *mcr-1* was chromosomally integrated. **Discussion**: The study area showed near-zero colistin use in livestock alongside high overall AMU, suggesting that the *mcr-1*-positive isolate may have evolved through co-selection rather than direct colistin pressure. *mcr-1* can persist and circulate in the environment, even where colistin use is minimal, and foxes may represent effective sentinels for tracking such events. Our results emphasise the importance of including wildlife in One Health surveillance frameworks to monitor AMR at the human–animal–environment interface.

## 1. Introduction

Antimicrobial resistance (AMR) is among the major threats to global health, driven by complex ecological processes across interconnected human, animal and environmental compartments [1], making it a pivotal One Health issue. The main driver of AMR spread remains the misuse and overuse of antimicrobials [2,3], while anthropogenic pressures, including urbanisation and environmental contamination by antimicrobial residues, may also contribute to its dissemination [4]. Wildlife exposed to contaminated ecosystems can acquire resistant bacteria and antimicrobial resistance genes (ARGs), facilitating their circulation across compartments [5]. Given this complexity, continuous surveillance and the early detection of emerging ARGs are essential within a One Health framework. While AMR surveillance programmes are common in human healthcare and food-producing animals [6,7], environmental surveillance remains comparatively underdeveloped [8]. This surveillance gap is becoming increasingly relevant, given that urbanisation and landscape transformation due to human activities are reshaping wildlife ecology [9]. Agricultural intensification, socioeconomic change and ecological fragmentation [10] are driving many wildlife species into peri-urban and urban settings. Increasing contacts between wildlife, livestock and humans facilitates cross-species pathogen transmission [9] as well as AMR spread [11]. Accordingly, the One Health Priority Research Agenda on Antimicrobial Resistance identifies wildlife and the environment as essential components of integrated AMR surveillance [2]. As wild animals are not usually exposed directly to antimicrobial treatments, they represent suitable indicators of environmental contamination [12]. The presence of AMR in wildlife reflects species and habitat characteristics [13]: opportunistic species that thrive in human-altered environments are more likely to carry and spread resistant bacteria [14], and resistant strains are more frequently found in animals living in anthropised areas [15]. This further supports the use of free-ranging synanthropic wildlife as sentinels for environmental AMR [16,17,18,19,20,21,22]. Among potential sentinel species, the red fox (*Vulpes vulpes*, Linnaeus, 1758) seems particularly suitable, as highlighted by several studies [23,24,25,26,27]. Its wide distribution, ecological adaptability and synanthropic behaviour expose this species to anthropogenic sources of AMR, such as waste and sewage [24,25,26].

While it is important to preserve the efficacy of all antimicrobials, some are considered more important than others. In particular, the World Health Organization (WHO) identifies some classes, used in both humans and non-human sectors, as highest-priority critically important antimicrobials (HPCIAs) for human medicine [28]. Colistin, for instance, is considered an HPCIA and remains an important treatment option for multidrug-resistant (MDR) Gram-negative infections, including those caused by *Escherichia coli* [5,29]. Aside from its clinical use in humans, colistin has been widely used in veterinary medicine, contributing to the selection and dissemination of ARGs, such as mobile colistin resistance genes (*mcr*), that may continue to persist in the environment [30]. These ARGs are usually carried in plasmids, which facilitate their rapid dissemination [31]. Although *mcr* genes have been sporadically identified in other wildlife species [32,33,34] and, more recently, in farmed foxes [35], their presence has never been detected in wild red foxes [24,27,36,37]. In this study, we report the first detection of a mobile colistin resistance gene (*mcr-1*) in free-ranging red foxes, carried by an MDR *E. coli* isolate.

## 2. Case Presentation

### 2.1. Sampling and Geographical Background

Between January and February 2026, 67 red fox carcasses (hunted, culled or found dead) were collected in the province of Brescia (Lombardy region, Northern Italy) and sent to the Brescia Diagnostic Section of the Istituto Zooprofilattico Sperimentale della Lombardia e dell’Emilia Romagna (IZSLER) for official diagnostic analyses, within the framework of the Lombardy Region’s Regional Wildlife Health Monitoring Plan (D.d.g. n. 13852 del 2021). In the province of Brescia, the control, management and hunting of foxes are governed by provisions specific to Alpine and lowland areas. These provisions are integrated with regional and provincial hunting calendars (d.c.p. di Brescia n. 45/2003, Regulation No. 16/2003). In addition to the routine diagnostics, faecal samples were collected as part of an ongoing pilot surveillance activity on AMR in wildlife.

**Figure 1:**
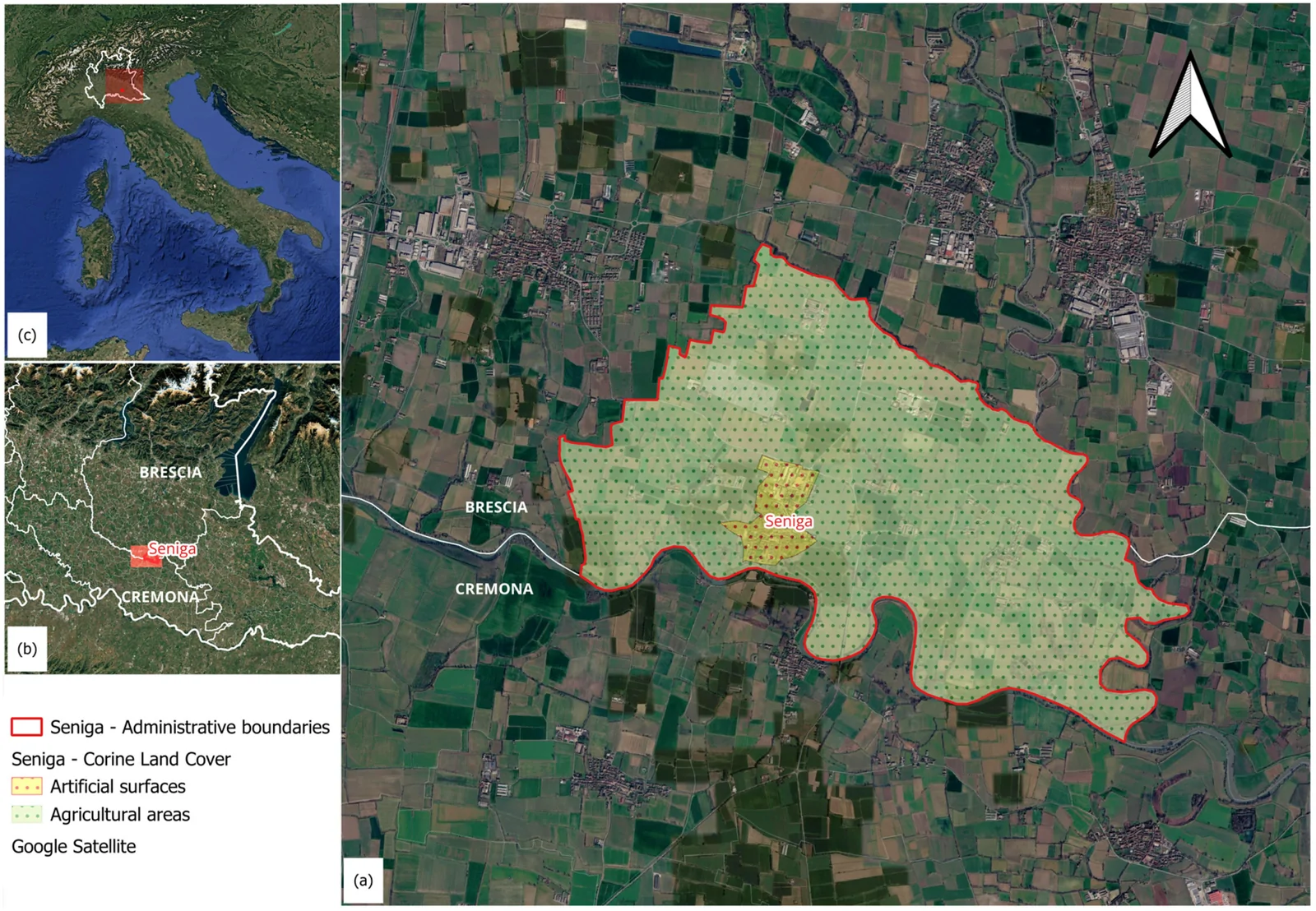
Geographic location and land cover characterisation of the study area: (**a**) A detailed land cover map of the municipality of Seniga, based on the CORINE Land Cover (CLC) classification, shows the artificial surfaces (yellow with orange dots) and agricultural areas (green with dark green dots) overlaid on satellite imagery; (**b**) a regional framework of Lombardy shows the administrative boundaries and highlights the provinces of Brescia and Cremona, as well as the municipality of Seniga; (**c**) the national location of the Lombardy region within Italy.

A single adult male red fox culled in February 2026 in the municipality of Seniga was found positive for an *Escherichia coli* isolate harbouring an *mcr* gene. The subject showed good nutritional status and no external signs of disease. The municipality of Seniga covers an area of 13.57 km^2^ and has a population of 1447 inhabitants, 11 cattle farms and one pig farm. It is located in the province of Brescia and borders the province of Cremona (Figure 1). According to the CORINE Land Cover classification (Figure 1), the municipality’s land use is predominantly agricultural, with arable land accounting for 96.2% (13.06 km^2^) of the total area. The remaining 3.8% (0.52 km^2^) consists of urban fabric (artificial surfaces).

### 2.2. Microbiological, Molecular and Genomic Characterisation

Following the microbiological protocol, 1 g of faeces was diluted 1:10 in 9 mL of buffered peptone water for pre-enrichment. After overnight incubation at 37 °C, a loopful (10 µL) of the enrichment broth was plated onto MacConkey agar supplemented with 1 mg/L Cefotaxime and onto ChromID^®^ CARBA SMART agar (bioMérieux, Marcy-l’Étoile, France). *Escherichia coli* was selected as the target bacterial species due to its ubiquity, the ease with which it can be isolated and the large number of existing studies on it. Positive growths were identified as pink to dark-pink colonies (lactose+). A single bacterial colony from the supplemented MacConkey agar (no growth was observed on the ChromID^®^ CARBA SMART agar) was selected for PCR-based *E. coli* phylogenetic group assignment [38] and ARG detection. Colonies were resuspended in 250 μL of DNase–RNase-free water, and DNA was extracted by lysis by boiling (98 °C for 10 min). The detection of resistance genes was first performed through a set of targeted PCRs. A multiplex PCR for the detection of extended-spectrum β-lactamase (ESBL) genes was set up using specific primers for *bla*_CTX-M_, *bla*_TEM_ and *bla*_SHV_ [39], and for *bla*_CMY_ [40]. Two multiplex end-point PCRs were set up to target *mcr-1* to *mcr-5*, and *mcr-6* to *mcr-10*, respectively, using previously published primers for the amplification of alleles 1 to 9 [41,42], and primers designed based on the first submitted sequence [43] for *mcr-10*. For the detection of carbapenemase-producing genes, a multiplex PCR was performed using specific primers for *bla*_VIM_, *bla*_SPM_, *bla*_IMP_, *bla*_NDM_, *bla*_OXA-48_ and *bla*_GES_ [44].

The *E. coli* isolate was subjected to antimicrobial susceptibility testing by determination of minimum inhibitory concentrations (MICs) with the Sensititre Vizion Digital MIC (Thermo Fisher Scientific, Waltham, MA, USA) using a commercial panel (Sensititre EUVSEC2 plate, Trek Diagnostics, Thermo Fisher Scientific^®^, Waltham, MA, USA) and a customised plate (Thermo Fisher Scientific). The latter panel included the following antimicrobials (MIC ranges expressed in mg/L): paromomycin (aminosidine) (1–32), amoxicillin–clavulanic acid (0.25/0.12–32/16), ampicillin (0.25–32), cefazolin (0.5–8), cefotaxime (0.5–4), colistin (0.03–8), enrofloxacin (0.015–32), florfenicol (1–64), flumequine (1–16), gentamicin (0.25–32), kanamycin (2–32), sulfisoxazole (sulfafurazole) (128–512), tetracycline (0.5–16) and trimethoprim–sulfamethoxazole (0.06/1.19–16/304). The isolate was classified as susceptible or resistant according to the European Committee on Antimicrobial Susceptibility Testing (EUCAST) epidemiological cut-offs (ECOFFs) [45].

The isolate was further sequenced on the Illumina MiniSeq platform. Libraries were prepared using the Illumina DNA Prep kit (Illumina, Inc., San Diego, CA, USA) according to the manufacturer’s instructions, and library quantification was performed with the Quantus Quantification System (Promega, Madison, WI, USA) using the QuantiFluor ONE dsDNA System kit (Promega). Raw read quality was assessed with FastQC 0.12.1, and reads were trimmed with trimmoamtic 0.39 using the following settings: LEADING:25 TRAILING:25 SLIDINGWINDOW:20:25 MINLEN:36, with adapter clipping. Trimmed reads were assembled with Unicycler 0.4.7, and the resulting assemblies were characterised with VirulenceFinder 2.0 [46], PlasmidFinder 3.0.3 [47], ResFinder 2.5.1 [48] and annotated with Bakta 1.12.0 [49]. Analysis of genome completeness was carried out with BUSCO 6.0.0 against the enterobacteriaceae_odb12 database [50], while contamination was assessed on the BV-BRC online platform using the Kraken2 database [51]. The location of *mcr-1* was assessed by aligning the whole 118 kb contig with BLASTn 2.17 (online version, core nucleotide database), and using MOB-suite 3.1.9 [52]. MOB-suite classifies assembly contigs based on the presence or absence of plasmid-associated replicons, oriT, relaxases and mate-pair formation markers, together with Mash-distance clustering against a reference plasmid database. Annotated contig comparison was visualised with Easyfig 2.2.5 [53]. All software tools were used with default settings unless otherwise specified.

### 2.3. Livestock Density and Antimicrobial Use Data Sources

Since the area was characterised by a high livestock density and a relatively low human population, data on livestock density and overall livestock antimicrobial use (AMU) (overall and colistin use) in the study area were compared with national, Nomenclature of Territorial Units for Statistics (NUTS) 2 (Lombardy), and NUTS 3 (Brescia and Cremona) levels. Livestock data were extracted from the Veterinary Information System dashboards [54], while AMU data were extracted from the ClassyFarm surveillance system [55] database. AMU surveillance is compulsory and fully covered by ClassyFarm, which integrates data from the National Electronic Veterinary Prescription System (REV). Over-the-counter antimicrobials for livestock are not available in Italy; farmers can only purchase them when prescribed by a veterinarian via the mandatory REV system [56]. AMU was expressed as Defined Daily Dose Animal for Italy (DDDAit) per kg of standardised livestock biomass (DDDAit/biomass); this indicator and its underlying metrics have been described in detail in previous studies [57,58,59]. Complete cattle data were available from 2021 to 2025. Detailed data on pigs could not be reported as only a single pig farm is present in the study area, precluding guaranteeing confidentiality.

### 2.4. Isolate Phenotypic and Molecular Characteristics

The *E. coli* isolate belonged to phylogroup F and carried both the *mcr-1* gene and an ESBL-encoding gene (*bla*_CTX-M_). It was phenotypically susceptible to colistin (Table 1), with a minimum inhibitory concentration (MIC) of 2 mg/L.

Antimicrobial susceptibility testing (Table 1) revealed an MDR pattern. Phenotypic resistance was observed for six β-lactams (amoxicillin + clavulanic acid, ampicillin, cefazolin, cefepime, ceftazidime, cefotaxime), as well as for enrofloxacin, ertapenem, gentamicin, florfenicol, tetracycline and trimethoprim + sulfamethoxazole.

Quality assessment of raw reads with FastQC revealed high sequencing quality, yielding 3,390,700 reads (505.2 Mb total) with a mean Phred score of 36. De novo assembly of trimmed reads resulted in 130 contigs with an N50 of 205,274 bp. Analysis of the assembled genome showed good completeness: C: 99.8% [S: 99.5%, D: 0.2%], F: 0.1%, M: 0.1 (where C = complete BUSCOs; S = complete and single-copy; D = complete and duplicated; F = fragmented; M = missing), and <2% contamination (0.3% no hits, 0.06% *Homo sapiens*, and 1% other). Total assembly length was 5.07 Mb, consistent with *E. coli* genome size.

Characterisation of the assembled genome identified AMR and virulence genes are reported in Table 2 and Table 3.

**Figure 2:**
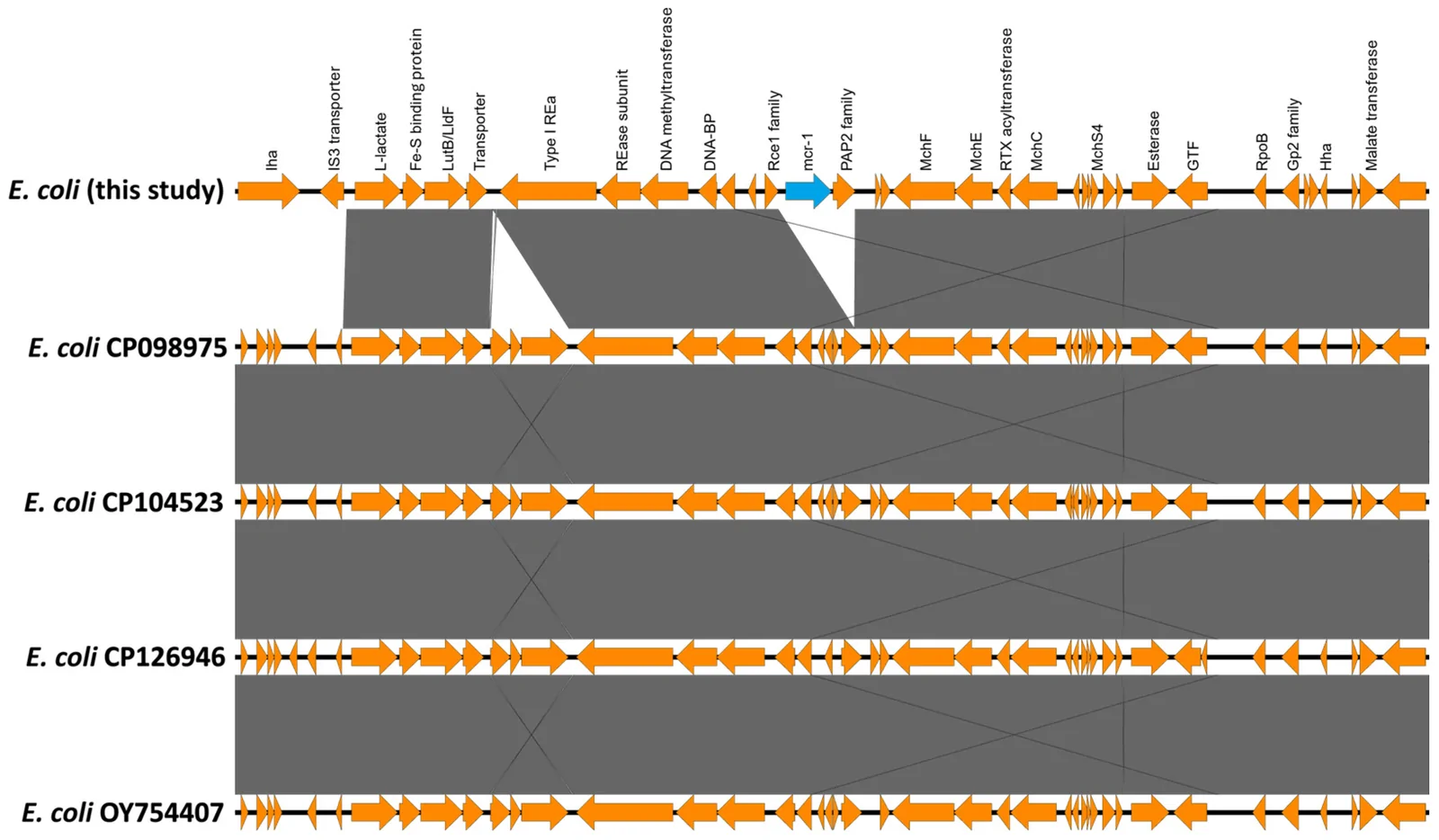
Easyfig comparison of the *mcr-1*-harbouring region with the four best BLAST matches. Dark grey areas indicate high similarity between sequences. For visualisation purposes, the genomic region displayed was limited to 20 kb upstream and downstream of *mcr-1*. Within a 118 kb contig, Bakta annotation revealed a 2606-nucleotide insertion carrying *mcr-1*, located between chromosomal genes involved in metabolism, oxidative stress response and iron transport. Specifically, the immediate genetic neighbourhood comprised an L-lactate-Rce1 family region and an Mch pathway/malate transporter region. This insertion showed 100% identity to an IncX4 plasmid sequence harbouring the same *mcr-1* allele along with the *pap2* gene. No ISApl1 element was identified in the genetic neighbourhood of the insertion.

PlasmidFinder identified the presence of a single IncY plasmid, which did not carry any resistance genes; BLASTn and MOB-suite analysis confirmed that the *mcr-1* contig lacked any plasmid markers and was therefore located on the chromosome (Figure 2).

### 2.5. Regional Comparison of Livestock Density and Antimicrobial Use

The cattle density in the study area (Seniga) was 483 heads/km^2^, 26.8 times higher than the Italian average (18 heads/km^2^), placing the municipality among the top 1% in the country. Cattle density at the NUTS 2 level (Lombardy) and NUTS 3 level (Brescia and Cremona) is reported in Table 4.

**Figure 3:**
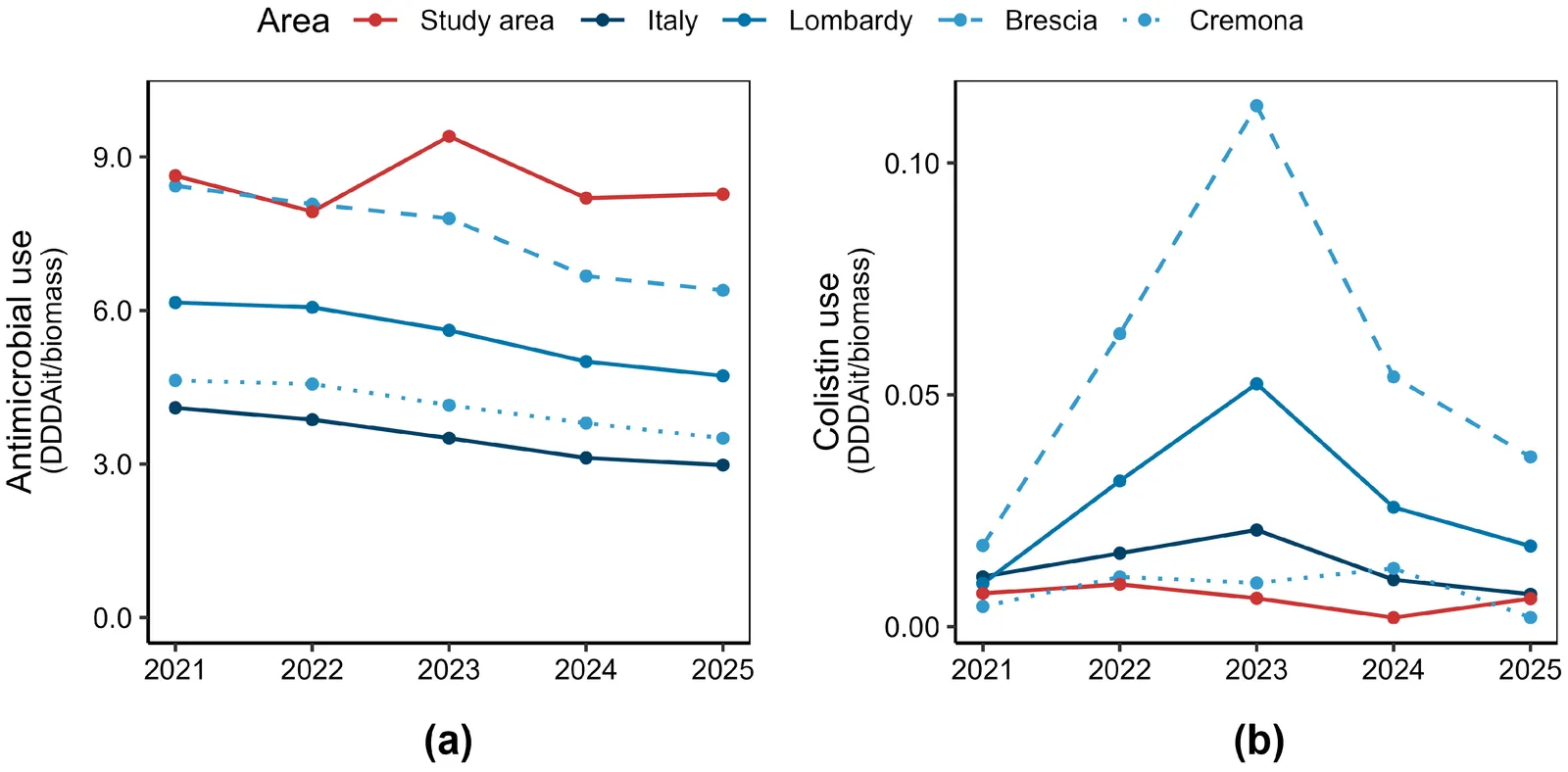
Trends in total antimicrobial (**a**) and colistin use (**b**) in cattle from 2021 to 2025 in the study area, the province of Brescia (NUTS 3), the neighbouring province of Cremona (NUTS 3), the Lombardy region (NUTS 2) and Italy. Antimicrobial use is expressed as Defined Daily Dose Animal per kg of standardised livestock biomass (DDDAit/biomass).

In the study area, the AMU in 2025 was 8.3 DDDAit/biomass, which was 177% higher than the national average (3.0 DDDAit/biomass). Colistin use was 0.006 DDDAit/biomass, which was 14.3% lower than the national usage (0.007 DDDAit/biomass). Total AMU and colistin use at the NUTS 2 (Lombardy) and NUTS 3 (Brescia and Cremona) levels are reported in Table 4, while Figure 3 shows the 2021–2025 trends.

No colistin was administered to pigs in the study area between 2021 and 2025.

## 3. Discussion

In this study, we report the identification of *mcr-1* in a faecal sample from a free-ranging fox. To the best of our knowledge, this ARG has never been described in wild red foxes before. The ARG was found in an MDR *E. coli* strain that also exhibited resistance to other classes of HPCIAs (i.e., fluoroquinolones, third- and fourth-generation cephalosporins) and was an ESBL producer. Although the isolate was still clinically susceptible to all tested carbapenems [60], its MIC was above the ECOFF for ertapenem. This class is authorised for human use only and is included in the WHO’s WATCH group [61], as well as the European Medicines Agency’s (EMA) Category A (avoid) [62]. The detection of a strain with such characteristics in the environment is of particular interest from a One Health surveillance perspective. The genomic characterisation of the strain was consistent with MIC results for nearly all tested antimicrobials; phenotypic resistance to streptomycin could not be confirmed as this antimicrobial was not included in the MIC panel.

Having established that *mcr-1* is chromosomally located in this strain, we further investigated the genomic context of this integration. Genome annotation revealed a 2606-nucleotide insertion comprising *mcr-1* between two chromosomal genes. This insertion is 100% identical to a region of an IncX4 plasmid (MW390532) carrying the same *mcr-1* allele, together with *pap2*, encoding a PAP2-family membrane-associated lipid phosphatase. This *mcr-1-pap2* cassette is frequently found on plasmids, typically flanked by ISApl1 elements. In our strain, however, these flanking transposases are absent, consistent with their loss after integration [63], despite being considered essential to the original mobilisation event. Although a different transposase, IS3, is present in our contig, its orientation and distance from the *mcr-1-pap2* cassette suggest that it was not involved in its integration, as transposases associated with *mcr-1-pap2* are typically located within 5 kb of the genes (e.g., [64,65,66]). A different scenario was observed for instance for the *dfrA14*-harbouring contig (trimethoprim resistance) where several transferases were observed. These two integration events likely occurred at different times: the presence of transposase/integron sequences in the *dfrA14* contig may be the result of a more recent acquisition, whereas the chromosomal segment comprising *mcr-1* may have had sufficient time (i.e., generations) to lose its flanking elements.

Virulence factors were consistent with an ExPEC (extraintestinal pathogenic *Escherichia coli*) strain. Numerous genes associated with adhesion (*fimH*, *papC*, *iha*, *fdeC*), iron uptake (*chuA*), host immunity evasion (*kpsE*), toxin production (*hlyE*) and bacterial competition (*mchBCF*) were detected in the isolate, underscoring its clinical and epidemiological relevance.

*mcr*-1 has been found in various sources, including human [67], food [68,69], environmental [70], and animal [71,72,73,74,75] samples. In foxes, this ARG has only been reported in one other wild species so far: the fennec fox (*Vulpes zerda*) [76]. More recently, it was also identified in *Klebsiella pneumoniae* strains isolated from captive foxes in China [35]. However, as these were farmed animals, the epidemiological implications likely differ from those of free-ranging foxes. In European wildlife, *mcr-1* has only been found sporadically. Specifically, it has been identified in several birds [13,77], while its presence in wild mammals has so far been limited to ungulates in Portugal [29,78].

Establishing the exact origin of the *mcr-1*-positive isolate remains challenging. Although the sampled fox was an adult, dispersal from a nearby area cannot be excluded, even though dispersal distances in this species are typically limited to around 10 km for male foxes [79]. The presence of this ARG in both livestock and humans in Northern Italy has been documented for over a decade [80,81]. Considering the high livestock density, extensive agricultural land use and relatively small human population in the study area, the isolate is likely to have originated from an animal source. Manure, together with shared water and food resources, can act as a vector for the transmission of ARGs between livestock and wildlife [82,83,84]. The exchange of ARGs is amplified when manure is used as a soil fertiliser and when wild animals defecate in these soils, creating a multidirectional network for the persistence and exchange of AMR determinants [3]. The presence of *mcr-1* in these compartments has previously been described in Northern Italy, where the ARG was frequently found in both manure and manure-fertilised agricultural soil [85].

The worldwide spread of *mcr* genes has been linked to the overuse of colistin in food-producing animals. This antimicrobial has been administered extensively in livestock for prophylactic purposes and is still used as a growth promoter in some countries [31,86,87]. However, following the discovery of the *mcr-1* resistance mechanism and its rapid dissemination, several countries have banned colistin as a feed additive [88]. In the EU, where antimicrobials had already been banned as growth promoters since 2006 [89], the EMA issued advice in 2016 setting targets for reducing colistin use in Member States [90]. In Italy, colistin sales were already in decline, falling by 98% between 2010 and 2021, which shifted the country from being one of the highest consumers in the European Union to one of the lowest [30]. Accordingly, the low colistin use observed in cattle in the study area and at all geographical levels, usually below 0.1 DDDAit/biomass (Figure 3), was consistent with the national decrease registered in previous years.

Since the fitness cost of *mcr-1*-carrying plasmids is high, reducing selective pressure could lead to a reduction in their circulation [88], as has been reported in Northern Italy over the years [91]. The characteristics of the *mcr-1*-positive isolate identified in this study may help to explain its persistence despite the near-absence of colistin use in the area. In particular, the chromosomal integration of *mcr-1*, which was confirmed by the contig topology, may have mitigated the fitness cost typically associated with plasmid-borne carriage, thereby reducing the reliance on active colistin selection for its maintenance. This is further supported by the lack of IS regions, which are crucial for the integration process and may have been lost after the integration occurred [63]. In addition, *mcr-1* was likely under-expressed, as suggested by its borderline phenotypic susceptibility to colistin (Table 1), which could have further reduced the burden of carrying the gene. The isolate was also resistant to several other antimicrobials (Table 1), including HPCIAs as well as penicillins, tetracyclines and sulphonamides, which are the most commonly used classes in European livestock [92]. Taken together, these findings suggest that the presence of this *mcr-1*-positive isolate may have been the result of co-selection rather than direct selective pressure from colistin use. Indeed, colistin use in the study area remained very low (<0.01 DDDAit/biomass) over the last five years, even though the total AMU was consistently higher than at the NUTS 2, NUTS 3 and national levels. The AMU in the study area also remained relatively stable between 2021 and 2025 (Figure 3), whereas other geographical levels showed a decline of 23–27%, suggesting there is still room for local AMU reduction. These results emphasise the importance of effective antimicrobial stewardship in reducing AMU, particularly in settings where ARGs can persist and circulate in the environment. Foxes inhabiting livestock-dense landscapes may represent effective sentinels for tracking such dynamics.

While our finding is suggestive of spillover of an *mcr-1*-carrying *E. coli* into wildlife from a livestock-dense, high-AMU environment, the nature of a single observation precludes drawing definitive conclusions. To establish a definitive origin of the isolate, concurrent sampling from local farms and environmental matrices (e.g., manure, soil and water) would be required, as manure used to fertilise fields in the study area may have originated from livestock housed in a different municipality. Although less likely given the characteristics of the study area, a human origin cannot be entirely excluded, for instance via contaminated water or wastewater. Therefore, future studies should combine longitudinal wildlife surveillance with comparative genomics of livestock, human and environmental isolates to establish direct transmission links.

A comprehensive understanding of AMR within a One Health framework requires surveillance systems that integrate data across humans, domestic animals and the environment. Genomic tools can further clarify the circulation of ARGs by enabling more direct tracing of transmission pathways, although their routine incorporation into surveillance systems may be constrained by costs and feasibility. Effective AMR surveillance also requires laboratory diagnostic findings to be interpreted alongside antimicrobial consumption data, as these two dimensions are inherently interconnected and should always be assessed jointly. Strengthening this integrated approach will be essential to anticipate and contain the emergence and spread of AMR.

## Figures and Tables

**Table 1:** Antimicrobial minimum inhibitory concentrations (MICs) of the *Escherichia coli* strain carrying the *mcr-1* gene. Where available, the European Committee on Antimicrobial Susceptibility Testing (EUCAST) epidemiological cut-offs (ECOFFs) for *E. coli* [45] of the tested antimicrobials are reported. MICs above the breakpoint are highlighted in bold and underlined.

Antimicrobial	Strain MIC (mg/L)	ECOFF Breakpoint (mg/L)
Paromomycin (aminosidine)	4	ND
Amoxicillin + clavulanic acid	**16**	8
Ampicillin	**>32**	8
Cefazolin	**>8**	4
Cefepime	**16**	0.125
Cefotaxime	**64**	0.25
Cefotaxime + clavulanic acid	≤0.06	0.25
Cefoxitin	8	16
Ceftazidime	**8**	1
Ceftazidime + clavulanic acid	≤0.12	1
Colistin	2	2
Enrofloxacin	**0.5**	0.125
Ertapenem	**0.06**	0.03
Florfenicol	**>64**	16
Flumequine	≤1	2
Gentamicin	**8**	2
Imipenem	≤0.12	0.5
Kanamycin	4	16
Meropenem	≤0.03	0.06
Sulfisoxazole (sulfafurazole)	>512	ND
Temocillin	8	16
Tetracycline	**>16**	8
Trimethoprim + sulfamethoxazole	**>16**	0.5

**Table 2:** Antimicrobial resistance genes detected by whole-genome sequencing (WGS) and the corresponding phenotypic resistance they confer in the *Escherichia coli* strain carrying the *mcr-1* gene.

Gene	Antimicrobial Resistance Encoded
*bla*_CTX-M-15_	Amoxicillin, ampicillin, aztreonam, cefepime, cefotaxime, ceftazidime, ceftriaxone, piperacillin, ticarcillin
*qnrS1*	Ciprofloxacin
*mcr-1*	Colistin
*tet(B)*	Doxycycline, tetracycline, minocycline
*aph(6)-Id*	Streptomycin
*aph(3″)-Ib*	Streptomycin
*sul2*	Sulfamethoxazole
*dfrA14*	Trimethoprim

**Table 3:** Virulence genes detected by whole-genome sequencing (WGS) and their associated virulence determinants identified in the *Escherichia coli* strain carrying the *mcr-1* gene.

Gene	Virulence Determinant
*aslA*	Not fully characterised
*air*	Enteroaggregative immunoglobulin repeat protein
*chuA*	Outer membrane hemin receptor
*csgA*	Curlin major subunit CsgA
*eilA*	Salmonella HilA homologue
*espY2*	Not fully characterised
*fdeC*	Intimin-like adhesin FdeC
*fimH*	Type 1 fimbriae
*hha*	Haemolysin expression modulator Hha (previous rmoA)
*hlyE*	Avian *E. coli* haemolysin
*hra*	Heat-resistant agglutinin
*iha*	Adherence protein
*kpsE*	Capsule polysaccharide export inner-membrane protein
*lpfA*	Long polar fimbriae
*mchB*	Microcin H47 part of colicin H
*mchC*	MchC protein
*mchF*	ABC transporter protein MchF
*nlpI*	Lipoprotein NlpI precursor
*papC*	Outer membrane usher P fimbriae
*shiB*	Homologs of the *Shigella flexneri* SHI-2 pathogenicity island gene shiA
*terC*	Tellurium ion resistance protein
*tia*	Tia invasion determinant
*yehA*	Outer membrane lipoprotein, YHD fimbriae cluster
*yehB*	Usher, YHD fimbriae cluster
*yehC*	Chaperone, YHD fimbriae cluster

**Table 4:** Cattle density, total antimicrobial use (AMU) and colistin use in the study area, and in the province of Brescia, the neighbouring province of Cremona, the Lombardy region and Italy. Nomenclature of Territorial Units for Statistics (NUTS) levels were reported where appropriate. The AMU refers to 2025 and is expressed as Defined Daily Dose Animal per kg of standardised livestock biomass (DDDAit/biomass).

Territorial Unit (NUTS Level)	Cattle Density (Head/km^2^)	Total AMU (DDDAit/Biomass)	Colistin Use (DDDAit/Biomass)
Study area	483	8.3	0.006
Brescia (NUTS 3)	97	6.4	0.037
Cremona (NUTS 3)	177	3.5	0.002
Lombardy (NUTS 2)	64	4.7	0.017
Italy	18	3.0	0.007

## Data Availability

The genomic dataset generated and analysed during this study has been deposited in the NCBI BioProject database under the accession number PRJNA1507251.

## Abbreviations

The following abbreviations are used in this manuscript:

AMR	Antimicrobial resistance
ARG	Antimicrobial resistance gene
WHO	World Health Organization
HPCIA	Highest-priority critically important antimicrobials
ESBL	Extended-spectrum β-lactamase
MDR	Multidrug-resistant
MIC	Minimum inhibitory concentration
NUTS	Nomenclature of Territorial Units for Statistics
AMU	Antimicrobial use
DDDAit	Defined Daily Doses Animal for Italy
EMA	European Medicines Agency
EUCAST	European Committee on Antimicrobial Susceptibility Testing
ECOFF	EUCAST epidemiological cut-off
